# Supplementary material for: Comparison of Pollutant Effects on Cutaneous Inflammasomes Activation
Source: Int J Mol Sci. 2023 Nov 23;24(23):16674. doi: 10.3390/ijms242316674 (PMC10706824; doi:10.3390/ijms242316674)
Supplement: Supplementary file 1 [file ijms-24-16674-s001.zip › ijms-2715208-supplementary.pdf]

Supplementary figure S1

1. **OXIDATIVE STRESS**

	Markers	DAY 1	DAY 4
Expression levels	4HNE	MP,UV,DEE,O_3	MP,UV,DEE
	SESN2	MP,DEE,CS,O_3,UV	O_3,CS,MP,DEE,UV

2. **DNA DAMAGE**

	Markers	DAY 1	DAY 4
Expression levels	8-OHdG	CS,DEE,UV,MP,O_3	UV,MP,DEE,CS,O_3

3. **SKIN AGING**

	Markers	DAY 1	DAY 4
Expression levels	MMP2	x	UV,CS,O_3,MP,DEE
	Type I Collagen	MP,DEE	DEE,UV,MP

4. **INFLAMMATION (INFLAMMASOMES ACTIVATION)**

	Markers	DAY 1	DAY 4
Gene expression	NLRP3	CS	UV
	NLRP1	O_3,CS	UV,CS,MP,DEE,O_3
	NLRC4	UV,CS	x
	NLRP6	O_3,CS	UV,MP,CS,DEE,O_3
Expression levels NLRs	NLRP3	DEE	x
	NLRP1	O_3,UV,CS,MP,DEE	CS,O_3,UV,DEE
	NLRC4	UV	x

	NLRP6	O_3,MP,CS,UV	O_3
Colocalization NLRs-ASC	NLRP3-ASC	x	DEE,MP
	NLRP1-ASC	MP,DEE,CS	O_3,UV,DEE,CS,MP
	NLRC4-ASC	x	x
	NLRP6-ASC	x	x
Protein expression levels	ASC	UV,CS,O_3,DEE	MP,CS,DEE,UV
	p30-Gasdermin D	O_3,UV, MP,	CS,UV,MP, O_3,DEE
	IL-1β	O_3,MP	UV,DEE,MP

Figure S1. List of the environmental pollutants affecting markers of oxidative stress, DNA damage, skin aging and inflammasomes components in human skin biopsies after one day (DAY 1) or 4 days (DAY 4) of exposure. The order of the environmental pollutants is listed in a decreasing manner, starting from the one with the major impact (red) to the one with the lowest effect on the mentioned markers. The "x" means no impact from any air pollutants.
